# Engaging rural women in healthy lifestyle programs: insights from a randomized controlled trial

**DOI:** 10.1186/s13063-015-0860-5

**Published:** 2015-09-16

**Authors:** Samantha L. Kozica, Cheryce L. Harrison, Helena J. Teede, Sze Ng, Lisa J. Moran, Catherine B. Lombard

**Affiliations:** Monash Centre for Health Research and Implementation, School of Public Health & Preventive Medicine, Monash University, Melbourne, Australia; Diabetes and Vascular Medicine Unit, Monash Health, Victoria, Australia; The Robinson Institute, Discipline of Obstetrics and Gynaecology, University of Adelaide, Adelaide, Australia

## Abstract

**Background:**

The obesity epidemic is well established, particularly in rural settings. Programs promoting healthy lifestyles for rural women are urgently needed; however, participant engagement is challenging. In the context of a large randomized controlled trial targeting the prevention of weight gain in rural women, we explored successful recruitment strategies and aimed to understand participants’ barriers, enablers and reasons for program participation.

**Methods:**

We recruited women (aged 18–55 years) from the general rural Australian population. A mixed-methods approach was applied to explore factors that influenced program participation, including quantitative questionnaires for all participants (*n* = 649) and qualitative semi-structured interviews conducted for a subgroup of participants (*n* = 45). Data were collected at three time points: baseline, 6 and 12 months post program commencement.

**Results:**

We recruited 649 rural women through a community communication and partnering strategy, a program marketing campaign and mobilization of social networks. Program participants were diverse across education and income levels and were representative of the wider Australian regional population. Factors that influenced program engagement were divided into personal (perceived program benefits and program accessibility) and social (peer persuasion and support). Identified enablers included convenience of the program location, perceived program utility, such as weight management and optimization of lifestyle choices, as well as attending the program with peer support. Barriers to engagement, which are likely exacerbated in rural communities included lack of anonymity, self-consciousness and segregated social networks in rural settings. Participants reported that eliciting local support and maximizing publicity is fundamental to improving future program engagement.

**Conclusion:**

Multiple program promotion strategies including communication, marketing and partnering, as well as mobilization of social networks and peer persuasion, enabled engagement of rural women into a healthy lifestyle program. These recruitment strategies are consistent with successful strategies utilized previously to recruit urban-dwelling women into lifestyle programs. Future engagement efforts in rural settings could be enhanced by hosting multiple sessions within existing socio-cultural networks and assuring participants that they will not need to share their personal health information with others in their community.

**Trial registration:**

Australia & New Zealand Clinical Trial Registry. Trial number ACTRN12612000115831. Date of registration 24 January 2012.

**Electronic supplementary material:**

The online version of this article (doi:10.1186/s13063-015-0860-5) contains supplementary material, which is available to authorized users.

## Background

The obesity epidemic is increasing globally and is a serious threat to public health. Socially disadvantaged populations such as those living in rural areas are at an increased risk of being overweight or obese compared with the general population. Higher rates of obesity in rural settings are associated with lower income and education levels, higher rates of unemployment and living in disadvantaged neighborhoods [1, 2]. Moreover, women living in rural settings are at the greatest risk of obesity in comparison to their urban counterparts [3, 4], with longitudinal data revealing reduced rates of physical activity and multiple barriers to achieving healthy lifestyles [4–6]. Despite the disproportionately high rates of obesity in rural populations, disadvantaged groups remain underrepresented in healthy lifestyle programs [7]. Efforts that encourage a healthy lifestyle are needed to address both the prevention and management of obesity in rural women.

The efficacy of healthy lifestyle weight gain prevention programs has been established [8, 9]. The potential advantages of prevention programs are that they may be more feasible to implement and require fewer resources and less funding with greater potential for population level impacts compared with the treatment of established obesity [10–12]. In support of obesity prevention, the World Health Organization (WHO) has named the prevention of weight gain as an international health priority [13]. WHO estimates that effective implementation of national-scale healthy lifestyle preventive programs has the potential to increase individual life expectancy by an additional 5 years [14]. The Australian Government has recognized the need to refocus health policies on prevention and established the National Preventative Health Taskforce (2009) to fund the implementation of healthy lifestyle programs. These healthy lifestyle programs have been implemented in community-based settings to promote physical activity and healthy eating [15]. To maximize the benefits of community-based healthy lifestyle programs, rigorous evaluation is vital and yet currently rarely completed [16].

Comprehensive program evaluation is required to inform scale-up through the exploration of implementation strategies and sharing of learning [17]. Assessment of program recruitment success and engagement is important in community evaluations [18] as recruitment and engagement can be challenging, especially within socially disadvantaged groups including those living in rural settings [19]. Low engagement [20] and retention rates are more pronounced among socially disadvantaged populations, resulting in labels such as “hard to reach” [21, 22]. Barriers to rural healthy lifestyle program engagement at the systems level include difficulty accessing the population, time constraints, cultural differences and limited knowledge [7]. Barriers at the participant level include multiple competing interests, distrust of researchers, lack of awareness of the research program and minimal public transport access [7, 23, 24].

Of the limited literature available, motivators for program engagement of disadvantaged populations include perceived personal benefits, minimal time commitments and program flexibility [25]. Social influences also have been reported to play a key role in engaging women [26]. However, the relationship between personal and social motivators in maximizing program participation remains unclear. To date, most literature exploring optimization of engagement has been conducted in populations with chronic diseases [27, 28], and few trials have been conducted in healthier populations or in rural areas. There is also a dearth of qualitative methods exploring engagement of healthy populations into lifestyle programs [29, 30]. In addition, most research in this area is based on researcher’s perceptions and experiences, rather than on those of participants [30]. Thus, consumer-driven evaluations are warranted. To address these identified gaps in the literature, we aimed to explore engagement, including strategies used to recruit women from small rural communities and their motivation for participation in a weight gain prevention program.

## Methods

### The HeLP-her Rural program

The Healthy Lifestyle Program (HeLP-her) is an evidence-based healthy lifestyle weight gain prevention program for women [8, 31]. The program has recently been implemented in rural communities (HeLP-her Rural) in an integrated community cluster randomized controlled trial (RCT) design [32]. This RCT aimed to prevent weight gain in a population of reproductive-aged women living in rural Victorian communities in Australia. The program was designed to be low intensity, low cost and non-prescriptive and focused on participants making small, long-termsustainable lifestyle changes. In this RCT 41 rural communities were randomized to the intervention (*n* = 21) or control (*n* = 20) groups.

### Program inclusion and exclusion criteria

Communities selected had a population size between 1500–10,000 people and were located 100–400 km from the capital city, Melbourne. Women aged 18–55 years of any BMI and residing in or close to participating communities were invited to participate. Exclusion criteria included pregnancy, or serious medical condition impacting program participation. The detailed study design methodology is comprehensively reported elsewhere [32]. This study was approved by the Monash Health Research Ethics Committee for research involving humans (project no.12034B). All participants signed informed consent.

### Recruitment strategies

Participant recruitment commenced in September 2012 and was completed in April 2013. A comprehensive communication and engagement protocol was developed to promote effective recruitment [32]. The recruitment strategy was deliberately simple and low cost to reflect usual practice and did not differ between the control and intervention communities. Women aged 18–55 living within each of the 41 selected communities were invited by letter and flyers to participate in this program. We engaged multiple levels of the community including: local government departments, health services, primary schools and kindergartens as well as community sectors (sports clubs, neighborhood houses and women’s groups) and community leaders.

Specific recruitment strategies incorporated:

1. Development of a multilevel partnering strategy across all local communities;
2. Flyers distributed to all families though partnership with the local primary schools, pre-schools and kindergartens within each town;
3. Staff presence and visits within the local towns promoting the program;
4. Media releases (newspaper and local radio);
5. Flyers distributed at additional locations (such as general practice and maternal child health clinics, pharmacies, community groups and town notice boards);
6. Mobilization of social networks through (a) encouraging women who had expressed interest and registered for the program to bring friends and family along to the program and (b) identifying community champions to promote the program to their community, workplace and social networks and accessing partners databases for email contacts.

Women expressed interest in study involvement by sending SMS text messages, phone, email or return of an expression of interest form.

### Program setting

The program was delivered predominately within each community’s local primary schools, and session times were scheduled to align with school drop off and pick up. In addition community locations such as local town halls, community centers and sports clubs were utilized for evening sessions to ensure multiple opportunities to participate. Sessions were able to accommodate young children.

### Measures of program engagement

We applied a mixed-methods approach to explore factors that influenced healthy lifestyle program recruitment and engagement. Here, we have defined engagement as factors that motivated, encouraged and enabled program participation, extending our definition beyond recruitment to include completion of the early phase of the HeLP-her Rural program (0–6 months). This phase included attending a single group education session, receiving SMS text messages, completing phone coaching and utilizing the program manual, described in detail in the study protocol [32]. We also highlight that a comprehensive process evaluation of the HeLP-her Rural program measuring reach, dose delivered and received at the individual level and program contextual influences has been described elsewhere [33].

### Data sources

Data were collected at three time points.

1. Baseline
  The baseline questionnaire included demographic, nutrition, physical activity information and a brief devised questionnaire exploring factors influencing healthy lifestyle program participation. The enablers and barriers investigated included questions related to social factors (attending a group, or individualized program, attending with friends and people you knew), program location (held in local town), program costs and program facilitator (local or external facilitator).
2. Six months post program commencement
  Qualitative methods (in-depth semi-structured interviews) were conducted with HeLP-her Rural participants. Purposeful sampling techniques were applied to obtain a representative sub-sample from the larger RCT. Women from 10 randomly selected communities (6 interventions and 4 control communities) were invited to participate in the semi-structured interviews and 65 women volunteered. Interviews were conducted until a point of data saturation, determined when no new ideas emerged from the interviews, as per standard methods [34]. One staff member conducted all interviews. The interview schedule topics included: (1) motivation for program enrollment, (2) speculated barriers preventing other community members from participating in this program and (3) recommendations to improve future healthy lifestyle program engagement. All qualitative semi-interviews were audiotaped and transcribed verbatim.
3. Twelve-months post program commencement
  The results of our semi-structured interviews informed the questions included in the 12-month questionnaire relating to potential community barriers to program engagement such as competing commitments, disinterest in health, self-consciousness and inconvenience of the program location and time. This enabled an opportunity to conduct data triangulation.

### Data analysis

Statistical analysis was performed using STATA version 12 for Windows (STATA, Texas, USA) and descriptive statistics used to explore participant demographic and anthropometric data at baseline (recruitment data). Continuous variables are presented as mean (SD) and categorical data as relative frequencies. Program retention data were assessed via Student’s t-test to determine whether there were differences in characteristics between participants that remained in the study (completers) compared to those that did not remain in the study (non-completers) at 12 months. However, categorical data (income, education and employment status) were compared between completers and non-completers using Fisher’s exact test because of the reduced numbers of participants within each category. Therefore, the categorical tests need to be interpreted with caution, as applying more robust statistical measures was not possible.

Qualitative transcripts were analyzed independently by two investigators via thematic analysis and key themes and patterns in the data identified. Thematic coding of data and development of models were assisted by the NVivo software program (QSR International Pty Ltd., version 10, 2012, Victoria, Melbourne).

## Results

### Participants

Quantitative sample: 649 women participated in this healthy lifestyle program. Baseline age and BMI in control (*n* = 301) and intervention (*n* = 348) participants were 39.6 ± 6.7 years and 28.8 ± 6.9 kg/m^2^, respectively, with no significant difference between groups (Table 1).

**Table 1:** Baseline characteristics of the overall study population

Participant baseline characteristic (*n* = 649)	
Age (years) mean (SD)	39.6 ± 6.7
BMI (kg/m^2^) mean (SD)	28.8 ± 6.9 kg/m^2^
Household income (AUD) *n* (%) 649	
≤$40,000	129 (23.0)
$41,000 − 64,000	115 (20.5)
$65,000 − 80,000	122 (21.8)
≥$81,000	195 (34.8)
Work *n* (%)	
Full time	108 (18.4)
Part time	317 (54.1)
Tertiary education *n* (%)	
No post-school qual.	110 (18.8)
Certificate	150 (25.6)
Diploma	105 (17.9)
Bachelor degree or higher	200 (34.1)
Country of birth *n* (%)	
Australia	545 (84.0)

Qualitative sub-sample: Based on purposeful sampling techniques employed, data saturation was met following 45 participant interviews (control and intervention) with a mean BMI of 30.25 ± 8.1 kg/m^2^ and age of 40.2 ± 4.4 years.

### Program recruitment

**Fig. 1:**
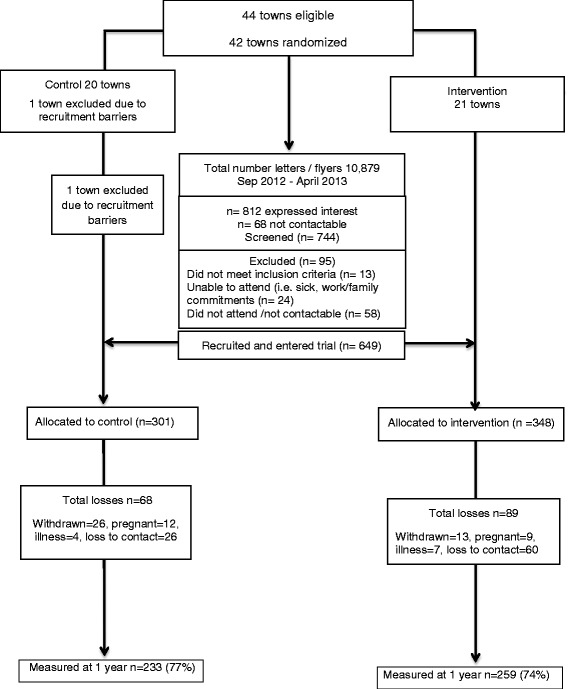
HeLP-her Rural CONSORT diagram

In total, 10,879 non-personalized program invitation flyers were distributed using the recruitment strategies outlined above. Overall, 812 women expressed interest in participation, and 649 women attended the program (80 %). This represented approximately 7-10 % of the eligible target population. Based on predefined and limited exclusion criteria, less than 12 % of women (*n* = 95) were excluded post screening. Reasons for exclusion included pregnancy, breastfeeding, taking weight control medication, diagnosis of a serious physical or psychological condition or not contactable (Fig. 1).

The women involved in this program were diverse across education levels, household sizes and income levels. Compared with the Australian Census information for regional-dwelling Victorian women of similar age (25–54 years), this suggested that the study cohort was representative of the wider Australian regional population, according to household income (median $47, 502AUD/year) and education (tertiary education reported in 30 %) [35].

### Enablers and barriers to engagement reported by the overall study population (quantitative)

**Fig. 2:**
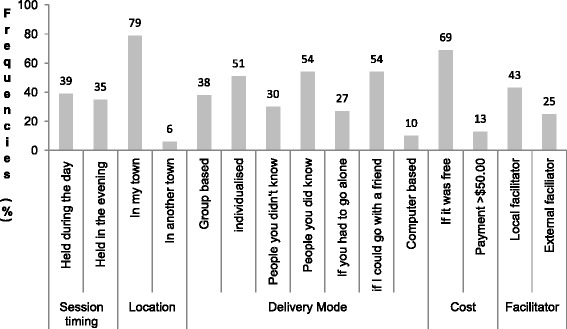
Factors encouraging program engagement reported by participants. Data collected at baseline (quantitative data); this provides an overview of the factors most likely to encourage program engagement reported by participants (control and intervention) at baseline. Factors have been grouped into five categories: session timing, location, delivery mode, cost and program facilitator. A higher frequency (%) reported indicates that a greater number of participants agreed that this factor would positively encourage program engagement

As there was no difference in demographic characteristics between those in the controls and interventions at baseline, results were pooled for all participants. Baseline results revealed factors influencing program engagement were related to the timing, venue and cost of the program, social influences as well as the mode of delivering the program (group based, individual, online). Program location (within women’s township) had the greatest impact on increasing the likelihood of participation and engagement (79 % agreeing with this statement), followed by the cost (69 % agreeing) and ability to attend the program with peer support (54 % agreeing). Conversely, few participants indicated that they would attend a healthy lifestyle program if it were held in another township (6 % agreeing), if the program was computer based (10 % agreeing) and if the program cost more than 50 dollars (13 % agreeing) (Fig. 2).

### Motivators for healthy lifestyle program engagement (qualitative)

**Fig. 3:**
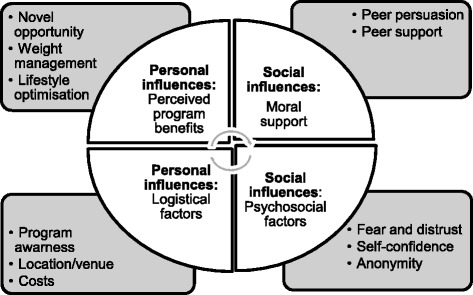
Motivators and barriers to healthy lifestyle program participation (qualitative data). This provides an overview of the motivators and barriers to engagement, which can be grouped into personal and social factors

Our thematic analysis of interviews revealed that the major reasons for participation in the HeLP-her Rural lifestyle program could be grouped into two broad categories: personal and social factors. Personal factors were divided into two sub-themes (1) perceived program benefits and (2) logistic factors associated with attendance. Social factors were also divided into sub-two themes: (1) peer support and persuasion and (2) psychosocial influences. Figure 3 portrays these themes.

### Theme 1: Personal motivators

- Perceived program benefits

Perceived personal program benefits were the strongest driver for program enrollment. Broadly participants described being motivated to participate for reasons related to weight management and to optimizing their health by improving their lifestyle choices. Participants frequently reported attending the HeLP-her Rural program as they were seeking external motivation to modify their lifestyle.

*“I actually needed to lose some weight and I thought something like that might motivate me to actually do it” (Control participant).*

Others reported attending the program to receive advice for weight-related conditions such as high-cholesterol, impaired glucose tolerance and polycystic ovary syndrome.

*“I was sort of on watch to manage my weight and change some habits to keep my blood sugar under control” (Intervention participant).*

However, participants also described enrolling in the HeLP-her Rural program for reasons unrelated to weight management. Women described joining the program because it was a unique opportunity and they were interested in women’s research.

“*There's nothing here [healthy lifestyle program] that I've heard about…there are a lot of myths, there’s a lot of this…but I really wanted to know how my body works”* (*Control participant).*

*“The research I found very interesting, yeah, because, I mean, it’s going to tackle obesity big time, hopefully, and improve things” (Control participant).*

(b) Logistical factors

Participants reported that their program involvement was aided by the *“ease”* and *“convenience”* of the program location, flexibility of session times and minimal participant burden. Hosting the program sessions at the local primary school was described as ideal as this venue was familiar to participants and enabled them to feel comfortable.

*“I think it was very appropriate, having it at the school because the school is a big part of our community” (Intervention participant).*

Participants also explained that as the sessions were run at school drop off and pick up times, this assisted program attendance as women *“didn’t have to make an extra effort*” to attend the program.

*“The program meetings were the right time of the day for me particularly but I imagine it’d probably be the right time of the day for most of the mums with children that are school aged” (Intervention participant).*

As the program was free this was also described as a motivating factor for program recruitment, allowing *“access to everybody.”*

Interestingly, while program awareness was described as high among program participants, lack of program awareness was speculated as a key barrier to greater program engagement. One woman explained that the program *“was well advertised, we had it almost every week in the school newsletter… and it was advertised in the [local] paper.”* On the other hand a participant from the same town reported, *“I think it was just possibly that there weren’t enough people that sort of knew it was actually happening.”* Participants described a range of other potential personal logistical barriers that may have prevented other women participating such as work, farming and family commitments, disinterest and limited access to childcare.

### Theme 2: Social influences

- Social support and peer persuasion

A strong relationship emerged between program engagement and the social dynamics and influences within rural settings. Members of participants’ social networks played a pivotal role in persuading and encouraging program involvement. The importance of program promotion through community members and social networks also resonated strongly.

*“[I went] because there were a couple of my friends going” and “word of mouth is always the best way in small towns” (Intervention participant).*

Moreover, participants described that attending the program with peer support boosted *“confidence”* and provided *“moral support.”*

*“A lot of people don’t want to go to these things (healthy lifestyle programs) by themselves; they might be uncomfortable and embarrassed” (Intervention participant).*

The need to attend a healthy lifestyle program with peer support was described as particularly important among obese women. Attending the program with friends reduced anxiety for program enrollment as there is *“safety in numbers.”* Indeed, almost all participants irrespective of their weight expressed preferring to attend a healthy lifestyle program with peer support rather than alone.

*“Amongst friends, amongst family,” you don’t feel out of place when you’re in a group” (Control participant).*

(b) Psychosocial factors

Lack of anonymity in rural settings emerged as a prominent barrier for greater community program engagement and uptake. Participants speculated that attending a healthy lifestyle program in a rural setting where you *“know everyone”* can result in women feeling *“self-conscious” and “embarrassed*” and can be a *“hurdle.”*

*“This is a small community—they’re nervous about doing something that everybody is going to be watching” (Control participant).*

The prospect of being judged by peers was exacerbated in obese women compared to lean women.

*“People are always self-conscious…do you want to admit that you want to lose weight in front of a whole heap of other mums at the school?” (Control participant).*

Others speculated potential psychosocial obstacles to program recruitment were distrust of a university-based program and *“fear of the unknown.”* Women revealing that there is a *“stigma”* attached with attending an urban, externally led community program.

*“People [are] apprehensive of what they are going to get into” and “what is going to be asked of them” (Intervention participant).*

Furthermore, a heightened sense of identity as well as place associated with community structures in rural settings was emphasized by participants. Participants revealed that while hosting a lifestyle program at the local government primary school was an ideal location for mothers of children at this school, this venue may have been “*off-putting”* and *“hindered”* and led to mothers of children from other schools feeling *“uncomfortable,"* discouraging program participation.

*“The schools are very segregated” and mothers of children from other schools may “have thought it (healthy lifestyle program) was just for our primary school” (Intervention participant).*

### Participant recommendations to improve future healthy lifestyle program engagement based on qualitative data collected

The participants described various strategies they believed would assist with improving future healthy lifestyle program engagement. These strategies were grouped broadly into four themes: (1) obtain community support via involving the community in the promotion of the program, (2) utilize multiple channels (written and verbal) to advertise the program in order to ensure maximum program awareness, (3) host multiple sessions within a range of rural community socio-cultural networks and (4) provide healthy lifestyle-related incentives (Table 2).

**Table 2:** Participant recommendations to improve future healthy lifestyle program engagement (qualitative data)

Recommendations	Explanations	Key quotes
Obtain community support and involve the community in the promotion of the program	• This will improve the social acceptability of the program being implemented	“I probably think if you had a local assisting…you’d have more of an (engagement). It’s like going to a Tupperware party. You go because, you know, you feel obliged”
	• If the program was promoted via a local community member this would increase community obligation to participate	
		“Word of mouth…if you could get hold of a couple of people beforehand to act as your agents, to try and rope people in”
Advertise the program regularly via multiple channels (written and verbal) to ensure women receive maximal exposure to the program promotion	• Many women reported needing to see and hear about the program multiple times prior to enrollment	“You know, it’s reading the flyer and reading it every day and seeing it… [then thinking]..I’ve got to go and do this and make time for it”
Host multiple program sessions within a range of socio-cultural settings and networks	• Holding numerous sessions at multiple venues will increase program reach among diverse social networks	“Maybe a few smaller sessions at the different schools”
		This will ensure people “don’t feel like [they] are going too much into somebody else’s territory”
Provide healthy lifestyle incentives (e.g., gym session, fruit platters and discounted gym memberships)	• May increase individual’s motivation to participate	“I was thinking maybe you could tee-up, like, a fitness session…beforehand or after the session, like a reward”
	• Provides an additional opportunity to promote healthy lifestyle	
		“You come to this [program], you get a free gym session”

### Potential barriers to healthy lifestyle program participation reported by the overall study population

Conducting data triangulation, we quantitatively explored potential barriers to program engagement by asking all program participants by questionnaire at 12 months to consider factors that may have prevented other women in their community from participating in the HeLP-her Rural program. The most consistently viewed barriers to greater program recruitment and engagement were associated with personal factors such as reduced program awareness (65 %) and work commitments (64 %). Social factors such as feelings of being embarrassed to attend the program were also reported by participants (40 %) (Table 3).

**Table 3:** Potential barriers to healthy lifestyle program recruitment reported by the overall study population (quantitative)

“Why do you think other women in your community did not join this program?”	
They were not aware of this program	298 (65 %)
Work commitments	295 (64 %)
Family commitments	228 (50 %)
Feeling self-conscious or embarrassed to attend program among your community members	180 (40 %)
Session time was inconvenient	133 (29 %)
They are not concerned by their weight	113 (25 %)
Venue was inconvenient	35 (7.5 %)

Results presented as relative frequencies (%)

### Program retention

At 12 months 74.5 % of the intervention and 77.5 % of control participants were retained (76.0 % overall). Reported reasons for missed data were censoring due to pregnancy, onset of a serious illness, relocation interstate, withdrawal and loss to contact. Overall, program attrition rates were higher among women with a heavier baseline BMI (BMI 31.7 kg/m^2^ versus 28.4 kg/m^2^) and a greater age (46.8 years versus 35.9 years). Other potential contributors including, household income, education and employment status were not associated with attrition. An additional file shows this analysis in more detail (Additional file 1).

Our 12-month retention strategy prior to review included: (1) sending invitational letters to all participants 2 weeks prior, (2) reminder phone calls made the week prior and (3) a text message sent the day prior. The invitation letter emphasized the low participant burden of the 12-month review with the invitation letter stating, “This review takes just a few minutes.” The review was scheduled at the same location as the baseline group session (local primary schools predominately) during school drop off and pick up to further minimize the participant burden. Additionally, successful retention strategies included flexible review schedules (evening, work and home reviews conducted as needed), frequent communication with local partners, rigorous tracking of participants, a skilled team and persistence. Gift packs were also provided to encourage women to return for the 12-month follow-up (Fig. 1).

## Discussion

This article reports on effective recruitment and engagement strategies employed in a community healthy lifestyle obesity prevention program within a rural context. Successful recruitment strategies included implementation of a community communication and partnering strategy, the use of several channels of program advertising and the mobilization of social networks. Our evaluation revealed factors that influenced program engagement could be grouped into two categories, personal and social factors. Personal factors related to perceived program benefits and program accessibility (logistics) and social factors were associated with peer support and persuasion. Additional social factors included the lack of anonymity in small rural communities, which may result in some women feeling apprehensive and too self-conscious to join a weight-related programs.

While the motivators for healthy lifestyle program enrollment have previously been established in obese women, which include improvements in physical appearance, self-esteem and confidence [36], there are no available qualitative studies exploring engagement in general rural populations. We addressed this research gap by conducting qualitative interviews in a population of healthy rural women as part of a consumer-driven evaluation [30]. Consistent with previous literature, we report that perceived program utility such as educational benefits improved the likelihood of program participation [25]. In addition, other drivers of HeLP-her Rural program engagement were weight management and optimization of lifestyle choices. We note that while we were able to recruit women across all BMI levels, obese women were more likely to enroll in the HeLP-her Rural program for weight management reasons, and lean women were more interested in lifestyle optimization. This provides a unique opportunity to understand the generalizability of the program and is important as current trends reveal that 20 % of healthy weight women will become overweight within 5 years, revealing a clear need to target women across the BMI spectrum [37].

Likely exacerbated in rural settings, our results indicated that social influences have an important role in both promoting and discouraging program participation in rural settings. Consistent with previous literature, we report that group-based programs promote opportunities for social contact and interactions [38] and that peer persuasion and encouragement improve healthy lifestyle program engagement. However, the lack of anonymity described in rural settings by our interviewees, defined as reduced opportunities for persons to have private and confidential areas of their lives [39], can discourage program engagement, particularly among overweight and obese women. Lack of anonymity issues are less likely to exist in urban settings as women have greater opportunities to participate in a wider range of weight-related programs and are unlikely to know everyone living within close proximity of their primary residence. To improve future healthy lifestyle engagement strategies in rural communities, we recommend emphasizing to potential participants that they will not be required to share their sensitive health information with other community members. This is hard to communicate via promotional flyers but could be achieved through verbal interactions or potentially through social media forums. Ultimately, the most feasible way to address lack of anonymity in small rural communities is to deliver a healthy lifestyle program remotely. However, the majority of participants here indicated they would not take part in a computer-based program with most preferring face-to-face delivery despite the associated limitations. Weight management programs utilizing face-to-face methods have also been found to be superior in preventing weight gain compared to web-based and correspondence programs [40, 41].

Interestingly, both our quantitative and qualitative results revealed that the greatest barrier to healthy lifestyle engagement was a lack of program awareness, despite the utilization of a multi-strategy communication and engagement approach. We report that participants required multiple personal verbal (peer persuasion) and non-personal visual (program marketing material) prompts prior to engaging with this program. Participants frequently reported that personal peer persuasion and word of mouth comprise the most valuable avenue to engaging rural women. Our results add to the findings of a recent systematic review indicating that increased awareness of a healthy lifestyle program among potential participants improves the likelihood of program engagement [42]. Further exploration on the number of times women need to view or hear about an upcoming healthy lifestyle program prior to enrollment is warranted. Additionally, exploration of the role of social media in engaging rural women into healthy lifestyle programs is needed as this low-cost strategy has been successful in urban populations [29]. Here, we focused our recruitment efforts on distributing promotional flyers via local schools and kindergartens and reached women with diverse socioeconomic backgrounds. However, some sections of the community such as the socially isolated disengaged community members may require more intensive engagement strategies [19].

Another important consideration when implementing healthy lifestyle programs in rural communities is the selection of an appropriate venue. As noted in other studies, the importance of ensuring cultural community sensitivity when attempting to gain entry for healthy lifestyle programs is vital [43]. In rural communities particularly, there appears to be a strong sense of place associated with various community venues such as the local government primary school, religious-based schools, and churches and/or specific sports clubs. For example, young people are not always familiar with hospitals or health venues, single women are not familiar with schools or kindergartens, and non-religious community members may feel uncomfortable attending a program held within local churches. The importance of coordinating health programs within a familiar environment has been shown to optimize engagement rates [44, 45]. Thus, we recommend hosting multiple group sessions within a variety of community structures even in small rural communities in order to reach diverse social networks. However, we acknowledge that this may increase total program costs, resources and staff time.

Despite young women being notoriously difficult to retain in healthy lifestyle programs [29], we retained 76 % of program participants at 12 months, which is comparable with previous studies [27, 29, 46]. Program retention strategies used within the HeLP-her Rural program focused on minimizing the participant burden via scheduling reviews at accessible locations and appropriate times in relation to the target audience. Building strong communication with partners, ensuring a skilled team, persistence and flexibility of review times improved our program retention consistent with a recent review [30]. Furthermore, the low intensity nature of the HeLP-her Rural program supported greater program retention in comparison to an intensive weight gain prevention trial, which reported a reduced program attendance rate of 50 % across multiple group meetings [47]. Thus, the utility and feasibility of intensive weight loss and weight gain prevention programs in young women appear poor with elevated attrition rates [47, 48].

We also report lower retention rates in women with heavier baseline BMIs, and this has been previously attributed to unrealistic weight loss expectations, leading to early disappointment and subsequent program drop out [49]. Our findings highlight the need to continually assess participant’s program expectations and to determine at baseline whether participants are indeed ready to commit to a lifestyle program to achieve weight management [49]. We further speculate that women with a higher age (47 years) were less likely to complete the HeLP-her Rural program as they may have had more intensive work commitment in comparison to the younger women (36 years) who likely had young children to care for at home. Future retention strategies may need to focus on older women with higher baseline BMIs.

### Strength and limitations

Strengths of the current study included the application of a mixed-methods approach to a rigorously designed healthy lifestyle prevention RCT. We also applied robust qualitative data analysis using a theoretical framework and utilized two independent staff for data analysis. Moreover, the purposeful sample utilized increases the generalisability of the qualitative results to the wider RCT cohort. In addition, it is unlikely that our results were influenced by differential recall bias at 12 months between intervention and controls as no statistical difference was reported between groups. Limitations include a lack of specific information regarding the impact and effectiveness of each individual recruitment strategy; therefore, we are unable to identify which engagement strategy was most effective in recruiting women from diverse socioeconomic backgrounds. Additionally, the barriers to engagement identified were speculative based on enrolled program participant’s views as we were unable to interview women who did not participate in this program.

## Conclusion

The HeLP-her Rural program provided a unique opportunity to explore participant engagement including recruitment and participation in a healthy lifestyle program specifically targeting rural-dwelling women. We engaged communities and rural women through multiple channels of communication (verbal and visual) in order to reach program recruitment targets. Our findings suggest that factors that need to be considered when engaging rural women into a healthy lifestyle program include using existing community social networks to deliver programs, promoting the program multiple times through local community networks, maximizing program awareness and understanding community social dynamics. Future program engagement would be enhanced by hosting multiple programs within existing socio-cultural networks and assuring participants that they will not be required to share personal health information with other women living in their community. Ultimately, the learning and practical implications reported in this study can be applied to future prevention programs more broadly, enhancing program engagement.

## Additional file

Additional file 1:
**The HeLP-her Rural program retention data analysis.** This provides the HeLP-her Rural program retention data analysis using ^†^Fisher’s exact test comparing the characteristics between program completers and non-completers at 12-months post-program commencement. (PDF 194 kb)

## Abbreviations

WHO: The World Health Organization
HeLP-her Rural: The healthy lifestyle program for rural women
RCT: Randomized controlled trial
BMI: Body mass index
