# Supplementary material for: Engaging rural women in healthy lifestyle programs: insights from a randomized controlled trial
Source: Trials. 2015 Sep 16;16:413. doi: 10.1186/s13063-015-0860-5 (PMC4574013; doi:10.1186/s13063-015-0860-5)
Supplement: Additional file 1: — The HeLP-her Rural program retention data analysis. This provides the HeLP-her Rural program retention data analysis using †Fisher’s exact test comparing the characteristics between program completers and non-completers at 12-months post-program commencement. (PDF 194 kb) [file 13063_2015_860_MOESM1_ESM.pdf]

Additional File 1: The HeLP-her Rural program retention data analysis

	n	Completers Mean(SD)	n	Non-completers Mean(SD)	p-values
**Weight (kg)**	577	77.4(18.5)	61	86.2(20.9)	0.0006
**Age (years)**	577	39.5(6.7)	4	46.8(2.8)	0.03
**BMI (kg/m^2)**	577	28.4(6.4)	57	31.7(7.8)	0.0003
**Income AUS (n%)†**					
**$40,000 or less**	-	122(22.34)		2(50)	0.39
**$41,000 to 80,000**	-	113(20.7)		-	
**More than $80,000**	-	311(57.0)		2(50)	
**Highest education†**					
**No post-school**		104(18.2)		1(25)	0.41
**Certificate/apprenticeship**		166(29.1)		2(50)	
**Diploma/Bachelor degree**		301(52.7)		1(25)	
**Occupation†**					
**Full time paid work**		104(18.2)		2(50)	0.22
**Part time**		311(54.5)		1(25)	
**No paid work**		156(27.3)		1(25)	

† Fisher’s exact test
